# Cancer Stem Cell-Associated Pathways in the Metabolic Reprogramming of Breast Cancer

**DOI:** 10.3390/ijms21239125

**Published:** 2020-11-30

**Authors:** Sara El-Sahli, Lisheng Wang

**Affiliations:** 1 Department of Biochemistry, Microbiology and Immunology, Faculty of Medicine, University of Ottawa, 451 Smyth Road, Ottawa, ON K1H 8M5, Canada; selsa056@uottawa.ca; 2 Ottawa Institute of Systems Biology, University of Ottawa, 451 Smyth Road, Ottawa, ON K1H 8M5, Canada; 3 Regenerative Medicine Program, Ottawa Hospital Research Institute, Ottawa, ON K1H 8L6, Canada

**Keywords:** breast cancer, cancer stem cells, metabolism, signaling pathways

## Abstract

Metabolic reprogramming of cancer is now considered a hallmark of many malignant tumors, including breast cancer, which remains the most commonly diagnosed cancer in women all over the world. One of the main challenges for the effective treatment of breast cancer emanates from the existence of a subpopulation of tumor-initiating cells, known as cancer stem cells (CSCs). Over the years, several pathways involved in the regulation of CSCs have been identified and characterized. Recent research has also shown that CSCs are capable of adopting a metabolic flexibility to survive under various stressors, contributing to chemo-resistance, metastasis, and disease relapse. This review summarizes the links between the metabolic adaptations of breast cancer cells and CSC-associated pathways. Identification of the drivers capable of the metabolic rewiring in breast cancer cells and CSCs and the signaling pathways contributing to metabolic flexibility may lead to the development of effective therapeutic strategies. This review also covers the role of these metabolic adaptation in conferring drug resistance and metastasis in breast CSCs.

## 1. Breast Cancer and CSCs 

Breast cancer is the most commonly diagnosed cancer in women, being among the top four cancers with the highest incidence rate [1]. Breast cancer can be divided into four distinct molecular groups based on gene expression analysis: Luminal A, Luminal B, human epidermal growth factor receptor 2 (HER-2)-enriched, and basal like breast cancer (BLBC). These subtypes are further characterized according to the expression of estrogen receptor (ER), progesterone receptor (PR), and HER-2 [2,3]. Luminal A accounts for 23.7% of breast cancer and is ER and/or PR positive, and HER-2 negative. Luminal B, on the other hand, accounts for 33.2% and is ER and/or PR positive, and HER-2 positive [4,5]. The majority of basal-like breast cancers lack the expression of ER, PR, and HER-2, in a subtype called triple negative breast cancer (TNBC), which accounts for 11.2% of breast cancers, is prevalent in young woman, and often relapses rapidly with poor prognosis [6]. The conventional treatment for a newly diagnosed tumor usually involves surgery, hormonal therapy, radiation, chemotherapy, and/or targeted therapy. However, drug resistance, recurrence, and metastasis create significant hurdles to recovery after treatment. Great effort has been made to decipher the pathophysiology and mechanisms underlying treatment resistance, metastasis, and disease relapse, attempting to develop better and more effective therapeutic strategies. Cancer stem cells (CSCs), a subpopulation of cells with tumor-initiating capabilities, have been suggested to play an important role in the poor clinical outcomes of breast cancer. CSCs are drivers of chemo-resistance, metastasis, formation of new tumors, and disease relapse [7,8]. Since their discovery, dysregulated signaling pathways associated with CSC survival and tumorigenesis have been revealed, providing potential therapeutic avenues to halt breast cancer development.

## 2. Metabolic Reprogramming in Breast Cancer and CSCs

**Figure 1:**
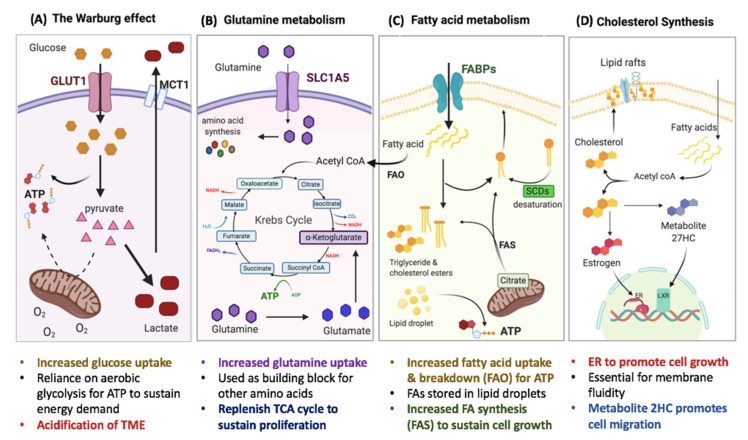
Metabolic adaptation in breast cancer cells and CSCs. (**A**) Cancer cells tend to adopt the Warburg effect or aerobic glycolysis, exhibiting a reliance on glycolysis instead of OXPHOS for ATP generation even with adequate supply of oxygen. The resulting pyruvate is converted into lactate and released outside the cell where it acidifies the tumor microenvironment (TME), creating an immunosuppressive environment. (**B**) Cancer cells also shift their dependence to glutamine for anabolic processes for cell growth (such as nucleotide and other amino acid synthesis) and to replenish the Krebs (tricarboxylic acid (TCA)) cycle. Glutamine is also important in the synthesis of glutathione, which is instrumental in chemo-resistance. (**C**) Both fatty acid oxidation (FAO) and fatty acid synthesis (FAS) are upregulated in breast cancer to supplement glycolysis for energy and to provide membrane materials for rapid cell proliferation and growth. (**D**) Cancer cells are also dependent on cholesterol synthesis for their membrane composition and signaling to promote growth and invasion.

It has long been recognized that cancer cells display a shift in their metabolic requirements to favor an increased glucose uptake relying primarily on glycolysis, even in the presence of adequate oxygen levels. This reprogramming, directing cancer cell metabolism towards aerobic glycolysis and away from mitochondrial oxidative phosphorylation (OXPHOS), is known as the Warburg effect (Figure 1A). Theories explaining this shift stem from the urgent need of cancer cells to increase cellular biomass and mass produce ATP to support their high proliferation rate as well as their need to compete with their surroundings [9]. Studies have since explored disrupting the Warburg effect as a way to halt tumor development and cancer cell survival, generating some pre-clinical success [10,11]. Interestingly, breast CSCs were shown to adopt a “Warburg-like” phenotype, indicating a link between metabolic reprogramming and tumor-initiation [11]. It is worth noting that while there is extensive support for the Warburg effect in aggressive types of breast cancer, certain subtypes of breast cancer and possibly even certain cell types within the same tumor tend to prefer OXPHOS for their energy supply [12]. 

In an effort to sustain their increased growth and proliferation, breast cancer cells also revamp their lipid metabolism, relying on de novo lipid synthesis to meet their energy demand and cell membrane synthesis [13]. Dysregulated lipid metabolism is characteristic of different types of breast cancer. Epithelial-like breast cancer cells were recently found to rely more on *de novo* fatty acid (FA) synthesis, while mesenchymal-like breast cancer cells had higher levels triacylglycerol (TAG) synthesis [14] and lipid droplets (LD) content, correlated with ‘stemness’ in several breast cancer cell lines [15]. Breast cancer cells could also shift their metabolism to favor fatty acid oxidation (FAO), where long-chain fatty acids are broken down supplying the tricarboxylic acid (TCA) cycle and OXPHOS to increase energy supply in the form of ATP, NADH, and NADPH in order to sustain cell growth (Figure 1C). Recent evidence suggests that aggressive CSCs, in particular, upregulate and rely on FAO [16]. Cholesterol content in cancer cells is linked to tumorigenesis, as it controls membrane fluidity in addition to acting as a signaling molecule for various cellular processes (Figure 1D) [17]. Recently, cholesterol was shown to be essential to breast CSCs [18]. Breast cancer cells could also increase their dependence on the amino acid glutamine to sustain cell proliferation by replenishing TCA cycle elements and by being a precursor to other amino acids (Figure 1B). Glutamine could also be converted to glutathione, which is linked to breast CSC enrichment and drug resistance [19,20]. 

Metabolic reprogramming is essential to the process of metastasis in breast cancer, where cancer cells spread to distant sites. A recent study suggests that different sites of metastasis require a different metabolic program. Liver metastatic breast cancer cells adopted a more glycolytic phenotype than breast cancer cells metastasizing to other sites [21]. Given the significance of metabolic reprogramming in tumorigenesis, chemo-resistance, and metastasis, more studies are now exploring the link between breast cancer oncogenesis, CSC pathways, and metabolic adaptation. 

Emerging evidence suggests that the pathways implicated in preserving the stem-like and self-renewal nature of CSCs are also implicated in metabolic reprogramming and metastasis. This review summaries several important CSC signaling pathways and their interplay with metabolic adaptations of breast cancer, as well as several potential therapeutic strategies. 

## 3. YAP/TAZ Signaling 

The Hippo pathway, with its major downstream effectors, Yes-associated protein (YAP), and transcriptional co-activator PDZ-binding motif (TAZ), is crucial in organ size control, development, proliferation, apoptosis, and differentiation [22,23]. Activation of Hippo signaling by mechanical cues or other pathways leads to the phosphorylation of YAP by a core of serine/threonine kinases, LATS1/LATS2 kinase. Phosphorylation of YAP then results in its accumulation in the cytoplasm, ubiquitination, and degradation [22]. However, hyper-activation of YAP/TAZ signaling has long been implicated in breast cancer tumorigenesis. A plethora of studies have associated YAP expression with poor prognosis in breast cancer patients [24,25,26]. Additionally, YAP is a key player in the survival of breast CSCs, correlating with higher levels of CSC marker (CD44) expression, and promoting the epithelial to mesenchymal transition (EMT) process as well as tumor metastasis [26,27]. Emerging evidence has made direct links between the YAP pathway and metabolic reprogramming in cancer cells, where different metabolic pathways regulate YAP activity and are also under the control of YAP.

### 3.1. YAP in Breast Cancer Metabolism

The relationship between YAP and glucose metabolism has been increasingly supported by multiple studies in different types of cancers [28,29]. In breast cancer, YAP/TAZ transcriptional activity was found to be controlled by glucose levels. A shift to aerobic glycolysis induced YAP while the knockdown of key glycolytic enzymes hampered YAP-induction in breast cancer. This regulation of YAP/TAZ in glycolysis-dependent cancer cells seems to occur through phosphofructokinase 1 [30]. Moreover, a side product of glycolysis, methylglyoxal, was found to increase YAP nuclear localization and activity by disrupting Hsp90 chaperone binding to LATS1, a kinase that inhibits YAP [31]. In a possible feedback response, YAP promotes glucose metabolism through the direct transcriptional upregulation of glucose transporter 3 (GLUT3), which is required for the increased shuttling of glucose into the cell required for aerobic glycolysis [32]. Additionally, cells with YAP-knockout exhibited a lower metabolic rate with reduced lactate production [33]. Therefore, the increased demand of glucose by cancer cells and the shift towards glycolysis could be aided and sustained by YAP activity in a feedback manner. YAP transcriptional activity also promoted cancer cell growth in a glutamine dependent manner. Blockade of transamination, where glutamine is converted to TCA elements, was shown to be more effective at suppressing breast cancer cells overexpressing YAP/TAZ, suggesting that YAP-promoting glutamine dependence is a crucial metabolic adaptation strategy in breast cancer [34]. 

YAP signaling has been implicated in several aspects of lipid metabolism, and their interplay has been studied. The enzyme Stearoyl-CoA Desaturase-1 (SCD1), which converts long chain saturated fatty acids to unsaturated fatty acids, has been established as a hallmark of CSCs in many cancers, including breast cancer [35]. A study by Noto et al. showed that SCD1 knockdown decreased the activity of YAP in CSCs, suggesting that lipid desaturation is essential for Hippo/YAP signaling. This regulation is thought to be partly mediated by the Wnt/β-catenin pathway, as Wnt ligands rely on unsaturated fatty acids produced by SCD1, and Wnt/β-catenin activation in turn stimulates YAP/TAZ activity [36]. Although this was shown in lung cancer, the same mechanism could also be essential for breast CSC survival due to their dependence on Wnt, YAP, and lipid unsaturation [27,37]. Interestingly, palmitic acid, an SCD1 substrate, was shown to inhibit YAP by inducing its phosphorylation through MST1 (an upstream YAP inhibitor along the Hippo pathway), leading to a reduction in endothelial cell stimulation and angiogenesis [38]. YAP is also implicated in fatty acid oxidation, where the fatty acid chains are broken down to generate ATP for cell growth. A recent study by Lee et al. found that tumor cells metastasizing to the lymph nodes required a metabolic shift to fatty acid oxidation, which occurred in a YAP dependent fashion [39]. This could be key in targeting breast cancer metastasis as lymph nodes are a common site for breast cancer cell spreading [40]. YAP directly interacts with the sterol regulatory element-binding proteins (SREBP1 and SREBP2), which are transcription factors that activate essential enzymes in fatty acid and cholesterol synthesis [41], further highlighting the extensive role YAP has played in metabolism. 

Cholesterol, as aforementioned, modulates the membrane fluidity of cancer cells and the self-renewal of breast CSCs [18,42]. Sorrentino et al. uncovered an overlap between YAP and cholesterol synthesis, where the mevalonate pathway (involved in cholesterol synthesis) led to the activation of Rho GTPases that in turn inhibits YAP/TAZ phosphorylation and degradation to increase YAP/TAZ nuclear localization and activity [43]. Statins, inhibitors of the rate limiting enzyme HMG-coA reductase in mevalonate metabolism, were shown to suppress YAP activation [43]. In order to better illustrate the role of the cholesterol pathway in breast cancer invasion and metastasis, Wang et al. found that the mevalonate pathway controlled RHAMM (receptor for hyaluronan-mediated motility) through direct transcriptional regulation by YAP in metastatic breast cancer cell lines [44]. Simvastatin was able to revert the invasive capacity of breast cancer cells by inhibition of the mevalonate/YAP/RHAMM axis [44].

### 3.2. Therapeutic Strategies for Targeting YAP-Mediated Cancer Metabolism

While YAP inhibitors are under development for the treatment of breast cancer, some already approved drugs have been repurposed to suppress YAP activity. Dasatinib, used in the treatment of chronic myelogenous leukemia, was found to inhibit the nuclear localization of YAP in breast cancer [45]. Table 1 provides some YAP inhibitors that have recently been in clinical trials. Since YAP interplays with cholesterol metabolism, drugs targeting key elements along the mevalonate pathway have garnered the most interest as YAP inhibitors. Zoledronic acid (ZA) that targets enzymes in the mevalonate pathway has been shown to inhibit YAP/TAZ activity [46] and was used in phase I–III clinical trials for the treatment of breast cancer (NCT00869206, NCT00566618, NCT03358017). Statins, common drugs used to lower blood cholesterol levels, were well tolerated and increased prognosis in breast cancer patients [47]. They are currently in clinical trials (NCT03454529, NCT02416427). A recent study has discovered that aspirin could overcome chemo-resistance in TNBC patients through the attenuation of YAP expression; the combination of chemotherapy with aspirin suppressed tumor growth in an in vivo animal model [48]. The effect of these YAP inhibitors on YAP-driven metabolic rewiring in glucose, glutamine, or fatty acid dependence remains a subject for further studies. 

## 4. The JAK/STAT Pathway

Janus kinases (JAK) are a family of intracellular non-receptor of tyrosine kinases, acting through their downstream effectors, the signal transducer and activator of transcription (STATs). JAK/STAT signaling is involved in various biological processes, ranging from immune responses to tumor development. JAK mediates the phosphorylation of STATs leading to their dimerization and nuclear translocation to activate various gene transcriptional programs [49]. STAT3 signaling is a well-known driver of breast cancer and CSCs, and is associated with worse relapse-free survival [49,50]. STAT3 is also widely implicated in drug resistance in different breast cancer subtypes [49]. Recently, JAK1/STAT3 signaling was shown to be essential in breast metastasis, where STAT3 was identified as the crucial mediator of breast cancer migration upon upstream JAK activation [51]. 

### 4.1. STAT3 in Breast Cancer Metabolism

STAT3 was shown to trigger the switch to aerobic glycolysis in mouse embryonic fibroblast cells [52], resulting in the upregulation of genes involved in glucose uptake and pyruvate formation while downregulating mitochondrial respiration, suggesting a role for STAT3 as a metabolic regulator. STAT3 was also shown to locate to the mitochondria and regulate its function, increasing growth and metastasis in breast cancer by protecting it from the cytotoxic effect of reactive oxygen species (ROS) [53]. Cells carrying a mutation in the mitochondrial localization sequence of STAT3 displayed a slower tumor growth rate [53]. Significantly, JAK/STAT3 is an essential mediator of lipid metabolism and fatty acid oxidation in breast CSCs, where it induces the expression of carnitine palmitoyltransferase 1B [54]. STAT3 was shown to promote breast CSC survival through FAO, where an FAO agonist rescued the breast CSCs from the inhibition of STAT3. Interestingly, glutamine was shown to activate STAT3 to induce cell growth and proliferation in breast cancer cells, indicative of an interplay/feedback loop between metabolic pathways and STAT3 activation [55]. This suggests that the inhibition of STAT3-mediated metabolic rewiring together with glutamine deprivation might be required to maximize treatment effect. 

### 4.2. Therapeutic Strategies Targeting STAT3

Considerable pre-clinical studies have investigated the inhibition of STAT3 in different cancers. Therapies were developed either by preventing the formation of STAT3-DNA complex or inhibiting JAK upstream elements. For example, a platinum (IV) compound called IS3 295 blocked STAT3-DNA binding properties to induce apoptosis in breast cancer [56]. Ruxolitinib, an FDA approved JAK1/2 inhibitor was found to attenuate STAT3 phosphorylation and reduce tumor growth in tamoxifen resistant breast cancer cells [57] and is currently under clinical investigation (NCT02876302, NCT02066532, NCT03012230). Table 1 shows some examples of direct and indirect STAT3 inhibitors currently in clinical trials for the treatment of breast cancer. 

## 5. PGC-1α Signaling Pathway

PGC-1α (peroxisome proliferator-activated receptor gamma coactivator 1) belongs to the family of transcriptional coactivators PGC-1 and is a well-known mediator of cellular metabolism in different tissues. PGC-1α has no DNA binding activity but stimulates gene expression by interacting with different transcription factors (e.g., NRFs, PPAR, ERRs) to regulate different aspects of metabolism, such as OXPHOS, glucose metabolism and fatty acid oxidation [58]. PGC-1α received increased interest in tumorigenesis and in the metabolic reprogramming of cancer cells. 

### 5.1. PGC-1α as a Regulator of Breast Cancer Metabolic Flexibility

Cancer cells exhibit metabolic flexibility, capable of regulating their metabolic needs and switching their reliance depending on environmental cues. Targeting one metabolic adaptation is insufficient, as tumor cells can re-direct their metabolism towards a different energy source. This agility enables cancer cells to grow beyond control. PGC1α was discovered as a master regulator of this metabolic flexibility in cancer, and its over-expression was associated with an increased bioenergetic flexibility of breast cancer cells [59,60]. Although the hyperactivity of PGC-1α in breast cancer is subtype dependent [61], PGC-1α has been linked to poor prognosis and metastasis in aggressive breast cancer [62]. Despite the documented role of PGC-1α in various oncogenic processes, its role in breast CSCs remains understudied. However, PGC1α is involved in the balance of pluripotency of stem cells [63,64] and is upregulated in pancreatic CSCs [65]. 

Various studies have elucidated the role of PGC1α in breast cancer metastasis. Circulating and invasive tumor cells may adopt a different metabolic program than the primary tumor cells. They could shift their reliance on mitochondrial biogenesis and oxidative phosphorylation. This phenomenon was shown to be mediated by PGC-1α, where metastatic cells adopting an EMT-phenotype were found to be reliant on the higher levels of PGC-1α to coordinate mitochondrial respiration and ATP production [66]. Andrzejewski et al. further showed that PGC-1α was a driver of breast cancer metastasis to the lung [60]. Treatment of metastatic cells with metformin (an inhibitor of complex I in mitochondrial respiration), however, was ineffective as the cells adopted a glycolytic phenotype. It was concluded that PGC-1α confers resistance to biguanides by increasing the bioenergetics capacity, and mitigates the switch between OXPHOS and glycolysis of the metastasizing cancer cells [60]. 

In addition to its modulation of the OXPHOS to glycolysis switch, PGC-1α is also associated with glutamine metabolism in ERBB2+ breast cancer cells; PGC-1α activity increased glutamine uptake and metabolism enzymes [67]. PGC-1α, along with ERRα (estrogen related receptor alpha), were also instrumental in reductive carboxylation, where glutamine is converted to citrate for anabolic processes to sustain cancer cell growth [67]. While there is little evidence regarding the role of PGC-1α in lipid metabolic reprogramming of cancer cells, a plethora of evidence and rationale suggest that PGC-1α could be a mediator for fatty acid uptake in different cell types, including adipocytes [68]. The breast cancer microenvironment is rich in cancer associated adipocytes (CAAs) that serve as “metabolic parasites,” supplying cancer cell with metabolites to enhance tumorigenesis [69]. The conversion of normal adipocytes to CAAs could possibly be mediated by PGC-1α. More work is required to elucidate the exact role of PGC-1α in the adaptations of lipid metabolism in breast cancer cells.

Through PGC-1α upregulation, RTK/Shc signaling was shown to drive the glucose dependence of HER2+ breast cancer cells, which leads to resistance to therapeutic effectiveness of phenformin (another biguanide targeting mitochondrial respiration) [59]. Inhibition of PGC-1α by shRNA- silencing resensitized breast cancer cells to phenformin and reduced their ability to withstand glucose withdrawal, suggesting an important role for PGC-1α in coordinating metabolic processes for cancer cell survival [59]. PGC-1α is also implicated in therapeutic resistance associated with the rewiring of tumor metabolism to protect against chemotherapy [70,71,72]. A study by Cruz-Bermúdez et al. showed that cancer cell lines resistant to cisplatin had an increased PGC-1α expression, and a shift to OXPHOS with an increase in mitochondrial mass [72]. They also found that cells with higher levels of PGC-1α had lower levels of cisplatin–induced apoptosis, further strengthening the link between chemo-resistance and PGC-1α. ROS is produced by the mitochondria in response to chemotherapy treatment [73], which confers resistance to the cells capable of adopting OXPHOS, and PGC-1α may play a role in this adaption. The correlation of PGC-1α with therapeutic resistance has also been documented in different tumors, such as ovarian, pancreatic, and colorectal cancers [70,71,74,75]. Taken together, these findings lead to the speculation that PGC-1α could also be a crucial player in breast CSC enrichment and chemo-resistance associated with metabolic agility, warranting further studies.

### 5.2. Therapeutic Targeting of PGC-1a in Tumorigenesis

Due to the importance of PGC-1α in maintaining cellular homeostasis, therapies targeting PGC1α activity are limited and could only be achieved through the inhibition its transcriptional coactivators (e.g., PPARs, NRF2, or ERRs) or the inhibition of the downstream metabolic pathways it instigates such as fatty acid or glutamine uptake. A study showed that ERRα regulates lactate metabolism in breast cancer, and ERRα antagonist Cpd29 was able to suppress breast cancer in vivo [76]. A natural compound*,* brusatol, has emerged as an NRF2 inhibitor, suppressing breast CSC enrichment and enhancing the effect of chemotherapy in TNBC [77]. Studies have emphasized the limitation of targeting these transcription factors alone due to the metabolic flexibility and adaptability of cancer cells [76,78]. Therefore, it is likely that combined therapies are required in developing an effective treatment. To date, there are no specific therapeutic drugs targeting PGC-1α, which remains an area of continuous research. Table 1 contains some potential approaches that could indirectly halt PCG-1α-associated metabolic reprogramming by targeting PPARa or ERRα. Lastly, a recent study showed that inhibiting ERRα exposes a metabolic vulnerability in breast cancer cells and increases the efficacy of glycolysis or glutaminolysis inhibitors [78].

## 6. HIF-1α Signaling 

Hypoxia inducible factor 1 (HIF-1) is a heterodimeric protein composed of two subunits, HIF-1α and HIF-β. During hypoxia, HIF-1α accumulates and dimerizes with HIF-1β to constitute an active transcription factor that binds to the hypoxia response element (HRE), leading to the expression of a wide range of genes involved in cancer progression, angiogenesis, metastasis, stem cell survival, cell growth, chemo-resistance, and metabolic rewiring. Over the last decade, HIF-1 has been shown to be stimulated by a multitude of different pathways that control HIF-1α, leading to the upregulation of HIF-1-mediated gene expression [79]. HIF-1α is often considered a hallmark for poor prognosis in breast cancer, with patients experiencing shorter distant metastasis- and disease-free survival [80]. In a recent study assessing the prognostic value of HIF-1α in 220 patients with stages II–III breast cancer receiving chemotherapy, HIF-1α expression was associated with a worse pathological response [81]. While studies have suggested a specific link between HIF-1α and hormone receptor+ breast cancers [82], HIF-1α/β protein expression and their transcripts are culprits in all breast cancer subtypes, particularly TNBC [83]. A study by Schwab et al. uncovered the essential role of HIF-1α in tumor initiation and breast CSC enrichment, and showed that deletion of HIF-1α decreased CSC enrichment and suppressed metastasis in vivo [84]. Other studies have shown that HIF-1α is a driver of drug resistance in breast cancer [85,86]. Its over-expression conferred resistance to chemotherapy of breast CSCs by inducing the expression of multi-drug resistance 1 (MDR1). This resistance was overcome by co-administration of an HIF-1α inhibitor and chemotherapeutics, preventing tumor relapse in vivo [86]. HIF-1α is also well documented in breast cancer metastasis, as it controls the expression of EMT-related genes and matrix metalloproteinases essential to cell migration [85,87].

### 6.1. HIF-1α in Breast Cancer Metabolism 

The role of HIF-1α in glucose metabolism has been extensively studied and established. HIF-1α acts as a transcription factor and directly induces the expression of glycolytic enzymes to contribute to the Warburg effect [88]. Intriguingly, HIF-1α controls the expression of pyruvate dehydrogenase kinase 1 (PDK1), a glycolytic enzyme that is involved in stem cell reprogramming [89]. In breast CSCs, hypoxia induces PDK1 to activate glycolysis and maintain stem cell characteristics [90]. NRF2, a regulator of stem cell self-renewal [91], was also found to mediate the HIF-1α induction of glycolysis-related genes [92]. NRF2 silencing in breast cancer reduced HIF-1α accumulation and metabolic rewiring [92]. Therefore, inhibiting the HIF-1α-mediated glycolytic switch could starve the tumor-initiating CSCs and reduce tumor development and progression. 

Bharti et al. analyzed the metabolic profiles of HIF-silenced tumors, and found that silencing of HIF-1α in TNBC decreased the levels of amino acids and shifted to glutamine dependence [93]. Mechanistically, it was shown that glutamate secreted by TNBC cells was derived from glutamine and HIF-1α activity was dependent on glutamine metabolism. In a paracrine fashion, the secreted glutamate enhances HIF-1α activity by depleting cysteine in cells, which is required for the ubiquitination of HIF-1α under normoxic conditions [94]. These two studies suggest the presence of a positive feedback loop in the HIF-1α mediated control of glutamine metabolism, where glutamine accumulation leads to further activation of HIF-1α. This could have therapeutic implications and should be considered when targeting cancer subtypes that are reliant on glutamine levels.

HIF-1α silencing in TNBC also influenced lipid signals and lipid droplets [93]. HIF-1α activity has been linked to the upregulation of fatty acid binding protein 7 (FABP7), which is involved in fatty acid uptake, binds to unsaturated fatty acids, and contributes to breast cancer tumorigenesis [95,96]. While studies insinuate that HIF-1α induces cells to increase their lipid uptake through FABP7 for energy production [95,97], more work is required to elucidate the role of HIF-1α in lipid metabolic alteration of breast cancer. 

### 6.2. Therapeutic Strategies Targeting HIF-1α

Given a well-established role of HIF-1α in tumorigenesis, some inhibitors have been developed to target HIF-1α in a wide variety of cancers. Vorinostat, an HDAC inhibitor suppressing HIF-1α by modulating its translocation [98], is currently in clinical trials for patients with breast cancer (NCT01720602, NCT03742245, NCT04190056). Digoxin, with preclinical success for the inhibition of HIF-1α and delay of tumor growth, is now in phase II of clinical trials (NCT01763931). Since several phase II clinical studies investigating HIF-1α inhibitors alone have been ineffective, a combinational therapy might be needed [99,100]. A combination of Vorinostat with tamoxifen was shown to reverse therapy resistance in a phase II clinical study [101]. Interestingly, a study analyzed the correlation of HIF-1α with prognosis and ascorbate (vitamin C) levels [102], and found that low grade tumors with lower HIF-1 pathway activation had higher levels of ascorbate [102]. This suggests that increasing vitamin C intake might reduce HIF-1-mediated cancer progression and increase patient disease-free survival. While the mechanism is still unclear, the antioxidant properties of vitamin C may quench the ROS that increases HIF-1α expression and activity [103,104]. Furthermore, vitamin C was suggested to be cytotoxic to cancer cells by inhibiting their energy supply through NAD depletion in the breast cancer cell line MCF-7 [105]. Together, these studies indicate that vitamin C is a safe therapeutic supplement either independently or through HIF-1α suppression to combat breast cancer metabolism. Table 1 lists several potential HIF-1α inhibitors used in breast cancer clinical trials. 

## 7. The NF-κB Pathway 

The NF-κB (nuclear factor kappa light chain enhancer of activated B cells) is a superfamily of transcription factors that can homodimerize or heterodimerize to control the expression of genes essential for cell survival and cytokine production. Activation of NF-κB signaling in breast cancer is well illustrated in tumorigenesis as it stimulates proliferation and inhibits apoptosis of cancer cells [106]. Certain subtypes of breast cancer rely on NF-κB activity more than others. NF-κB signaling has been associated with the loss of ER expression and is more commonly found in basal-like subtypes, such as TNBC [107,108,109]. Furthermore, ample evidence over the years has linked NF-κB signaling to breast CSC survival. NF-NF-κB is crucial to the expression of stem cell regulators, directly induces the expression of CSC marker CD44, and intersects with several pathways controlling self-renewal [110,111,112]. NF-κB signaling is also involved in chemo-resistance, and its inhibition has been shown to increase chemo-sensitization in breast cancer [113,114]. 

### 7.1. NF-kB in Breast Cancer Metabolism 

NF-κB as a player in the regulation of glycolysis has been documented in several cancer models. Inhibition of NF-κB was found to reduce aerobic glycolysis [115,116,117]. Mechanistically, NF-κB signaling induces a Warburg phenotype by upregulating the glucose transporter GLUT3 [118] and hexokinase [115] in cancer cells. In breast cancer, it was shown that NF-κB stimulated pyruvate kinase M2 (PKM2), which is a crucial enzyme in the last step of glycolysis [119]. The role of NF-κB in promoting the Warburg effect might be more complicated and beyond glycolysis, as it is also involved in the regulation of mitochondrial function. NF-κB could forgo mitochondrial respiration in favor of aerobic glycolysis in cancer cells. Johnson et al. found that the NF-kB element p65 translocated to the mitochondria in the absence of the NF-kB element p53, where it binds to mitochondrial DNA and suppressed gene expression, decreasing oxygen consumption and ATP levels [120]. Furthermore, metabolic reprogramming mediated by mitochondrial dysfunction and NF-κB activation in tumor-associated fibroblasts promoted breast cancer growth via a paracrine interaction [121]. TNBC exhibits a higher frequency of mitochondrial defects than other subtypes of breast cancer. Due to TNBC tumors showing a higher level of NF-κB signaling, this suggests a role for NF-κB in the switch from mitochondrial-dependent respiration to glycolysis, and in the metabolic reprogramming of TNBC cells. NF-κB has been shown to promote the acidification of the tumor microenvironment, which contributes to the hindered anti-cancer immunity in breast tumors [122,123]. 

While the effect of NF-κB on lipid metabolic adaptation in breast cancer remains unclear, there is evidence supporting the interplay of lipid metabolism and NF-κB activity. Studies have shown that NF-κB is dependent on and controlled by various elements of lipid metabolic pathways. Fatty acids were shown to induce NF-κB activity through the activation of TLR4 signaling [124]; fatty acid-binding protein 5 (FABP5) increased tumorigenesis of breast cancer cells possibly through the induction of NF-κB [125]; and the products of SCD1 were shown to be essential to NF-κB activity in ovarian CSCs [126]. More work is required to elucidate the mechanisms by which NF-κB is involved in lipid metabolism of breast cancer cells and CSCs. 

The role of NF-κB in glutamine metabolism and dependence in breast cancer has been somewhat established. In HER-2+ breast cancer, NF-κB was proven to be essential in the HER2-stimulated glutaminase expression, which is a rate-limiting enzyme involved in the metabolism of glutamine and its conversion to glutamate, leading to ATP provision and anabolic processes [127]. Additionally, Reid et al. revealed an essential role of IKKβ (an element along the NF-κB pathway) in glutamine metabolism as a glutamine level sensor [128]. IKKβ deficiency was found to increase the glutamine dependency of cancer cells, suggesting a possible therapeutic strategy by co-inhibition of both IKKβ and glutamine metabolism to reduce TNBC cell viability [128]. 

### 7.2. Therapeutic Strategies Targeting NF-κB Elements

Various NF-κB inhibitors have been developed, with some of them moving to clinical trials. Recent clinical trials have been investigated curcumin, that had shown some pre-clinical success [129,130,131], in newly diagnosed breast cancer patients (NCT03980509, NCT03847623) and chemotherapy-treated breast cancer patients undergoing radiotherapy (NCT01740323). Another phase I/II clinical trial of breast cancer involves Imx-110, a nanoparticle encapsulating the STAT3/NF-κB/poly-tyrosine kinase inhibitor and doxorubicin (NCT03382340). More recently, Baicalin (a natural compound) was found to be effective against breast cancer by downregulating lactate production, glucose uptake, and glycolytic proteins through the inhibition of the NF-κB/c-Myc pathway. Intriguingly, Baicalin inhibited tumor growth in vivo without showing significant side-effects, paving a way for potential clinical translation [132]. Table 1 lists several promising NF-κB inhibitors in recent clinical trials. 

## 8. The Wnt/β-Catenin Pathway 

Another important pathway in breast cancer progression and breast CSC survival is the Wnt/β-catenin pathway. The canonical Wingless (Wnt) pathway is activated when Wnt ligands released in the tumor-microenvironment bind to surface receptors to prevent β-catenin from phosphorylation and degradation of the β-catenin destruction complex (GSK3, APC, and Axin2), allowing for nuclear translocation of β-catenin and subsequent translational activity [133]. Wnt/β-catenin signaling is important in embryonic development, normal breast stem cell function, and tissue homeostasis. Its dysregulation is a hallmark of many cancers, in particular colorectal cancer, where a high frequency of APC mutation is observed [134]. In addition, hyper-activation of Wnt signaling has also been documented in breast tumorigenesis [135,136]. The Wnt pathway is considered a driver of breast CSCs, as β-catenin mediates the transcription of several genes crucial to breast CSC survival and self-renewal such as *SOX9* and *ALDH* [137]. Inhibition of Wnt/β-catenin signaling suppresses CSCs and reduces breast cancer metastasis and chemo-resistance [138,139]. 

### 8.1. Wnt in Breast Cancer Metabolism

Similar to NF-kB, Wnt signaling is also involved in the regulation of mitochondrial function and aerobic glycolysis [140,141,142]. Wnt/β-catenin signaling is thought to promote the Warburg effect in breast cancer through the inhibition of cytochrome c (an essential component in OXPHOS) [141] and through the expression of c-Myc [143]. A recent study by Wang et al. found that protein caveolin-1 inhibited aerobic glycolysis in breast CSCs by promoting the degradation of c-Myc, which is known to regulate the expression of glycolytic enzymes [144,145]. Yang et al. found that MCL (myeloid cell leukemia 1, a regulator of mitochondrial function and an anti-apoptotic protein) was controlled by Wnt5B [140]. The knockdown of Wnt5B (a protein in the non-canonical Wnt pathway independent of β-catenin) reduced MCL and suppressed mitochondrial OXPHOS, suggesting a role of non-canonical Wnt signaling in mitochondrial respiration [140]. This illustrates that Wnt signaling in breast cancer cells could lead to either an increase in glycolysis or OXPHOS to provide increased bioenergetics for the tumor. Whether these two pathways are preferentially turned on in specific cell types within the same tumors or in different subtypes of breast cancer remains an open question. C-Myc could also be involved in the shift of breast cancer metabolism towards a reliance on glutamine. C-Myc was shown to promote glutamine metabolism in several breast cancer cell lines by upregulating a glutamine transporter as well as the glutamine processing enzyme, glutaminase [146]. Lastly, Vergara et al. found that β-catenin knockdown increased de novo lipid synthesis enzymes, suggesting that Wnt signaling may suppress fatty acid synthesis [142]. 

### 8.2. Therapeutic Targeting of Wnt Elements in Breast Cancer

Given the role of Wnt signaling in cancer progression and CSC survival, many Wnt inhibitors have been developed and several are in clinical trials. ICG-001, a small molecule inhibitor that inhibits the interaction between β-catenin and its co-activator in the nucleus, has been in clinical trials for several cancers (NCT01764477, NCT01302405, NCT01606579). Vantictumab, a monoclonal antibody that binds to the FZD receptors to block Wnt binding, has recently been used in a phase I clinical trial to combat metastatic breast cancer. Similar to STAT3, glutamine deprivation was found to reduce β-catenin activity [147], suggesting that an effective approach to mitigating Wnt-induced metabolic rewiring hinges on glutamine management as well. Future therapies may need to take into account the effect of glutamine availability on pathway inhibition. Several Wnt inhibitors in clinical trials combating breast cancer are listed in Table 1.

## 9. Pathway Crosstalk in Metabolic Reprogramming

**Figure 2:**
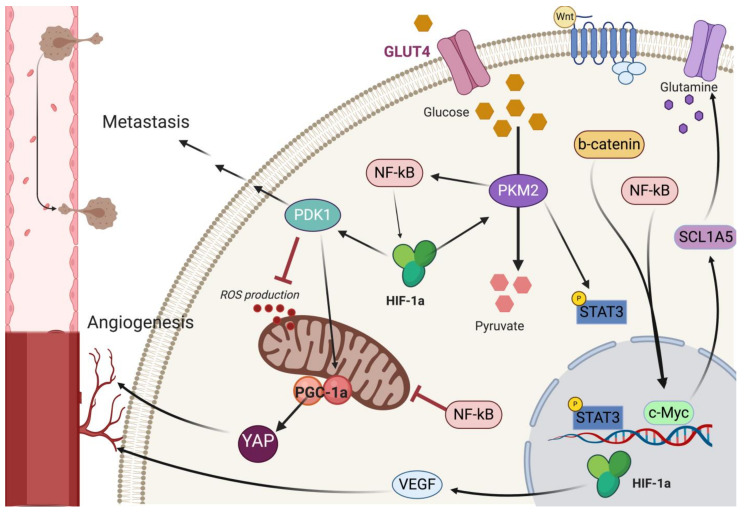
The intersection of CSC pathways and their modulation of metabolic adaptation. HIF-1α activates PDK1 (pyruvate dehydrogenase kinase 1), which promotes a glycolytic phenotype and increases cell migration and metastasis. HIF-1α in turn is controlled by Pyruvate Kinase M2 (PKM2) through NF-kB signaling. PKM2 also activates STAT3 driven gene expression. PDK1 induced by HIF-1α could also influence PGC-1α levels while reducing ROS production. YAP and PGC-1α work together to promote angiogenesis. Wnt/β-catenin and NF-kB both promote c-Myc expression, which induces glutamine dependence as it increases the expression of glutamine transporter SCL1A5. Note: pathways are simplified with only key elements shown.

All aforementioned pathways are interconnected in breast cancer progression and metabolic adaptation (Figure 2). This could have huge implications in therapeutic targeting of breast cancer and CSC metabolic rewiring, since the inhibition of one pathway alone could lead to compensation by the other pathways. 

HIF-1α was shown to upregulate STAT3 and increased its DNA binding and target gene expression, maintaining the CSC phenotype in TNBC [170]. In aerobic glycolysis, HIF-1α and STAT3 seem to be interconnected through PKM2 (pyruvate kinase M2), an enzyme in the final stage of glycolysis, leading to the production of pyruvate. PKM2 acts as a kinase phosphorylating STAT3 to activate STAT3 target genes [171]. PKM2 forms a positive feedback loop with HIF-1α [172]. Therefore, HIF-1α mediating PKM2 to control STAT3 could be an essential driver of the Warburg phenotype in breast CSCs. Inhibiting only one of these players might be insufficient to abrogate this metabolic adaptation. 

PKM2 is also involved in the interplay between HIF-1α and NF-κB. The regulation of HIF-1α by PKM2 seems to be NF-κB-dependent. PKM2 was shown to translocate to the nucleus and associate with NF-κB elements to promote HIF-1α-driven VEGF release [173]. Inhibition of PKM2 may potentially revert metabolic reprogramming as well as downregulate NF-κB and HIF-1α signaling. A PKM2 inhibitor, shikonin, has been shown to inhibit tumor growth and aerobic glycolysis, and sensitize cancer cells to chemotherapy treatment [174,175]. Moreover, the aberrant expression of the enzyme transglutaminase promoted metabolic rewiring and aerobic glycolysis through the constitutive activation of NF-κB that in turn activated HIF-1α signaling [176]. The pharmacological modulation of transglutsminase was shown to inhibit the malignant phenotype in breast cancer cells as well as cancer stem cells [177].

In liver-metastasis prone breast cancer cells, HIF-1α triggers the glycolytic phenotype required for migration by directly expressing the enzyme pyruvate dehydrogenase kinase 1 (PDK1) [21]. Of note, PDK1 knockdown leads to a downregulation of PGC-1α, as well as an increase in mitochondrial disorder and an increase in ROS production [178]. HIF-1α may upregulate PDK1, which promotes PGC-1α activity to reduce cancer cell damage caused by ROS accumulation. This machinery could be exploited by breast cancer cells to increase cell survival and overcome ROS-mediated cytotoxicity (Figure 2). Targeting PDK1 might serve as another therapeutic strategy to revert HIF-1α-mediated PGC-1 activity. Indeed, recent studies have highlighted the significance of PDK1 inhibition to increase the efficacy of other therapies on some cancer models [179,180,181]. The effect of the co-inhibition of PDK1 on the growth and metastasis of breast cancer is still open to investigation. 

Wnt and NF-κB signaling pathways are known to interact and regulate each other in breast cancer [182]. Chemotherapy-induced TNBC cells to release cytokines that activated both NF-κB and Wnt/β-catenin pathways, forming an autocrine forward-feedback loop to enrich drug-resistant TNBC bulk cells and CSCs [114]. Since both Wnt and NF-κB play roles in mitochondrial function [120,183], they could also modulate each other to affect metabolic rewiring through the mitochondria. Additionally, Wnt and NF-κB may work together to promote the Warburg effect and glutamine dependence as both pathways upregulate c-Myc [143,184]. Thus, an effective therapy would have to target both. Interruption of YAP signaling was found to suppress glycolysis, mitochondrial biogenesis, angiogenesis, and PGC-1α expression. PGC-1α knockdown, in turn, inhibits YAP-mediated endothelial cell activity and angiogenesis [185]. This provides a possible role for the PGC-1α/YAP axis in regulating metabolism and angiogenesis in breast cancer.

## 10. Concluding Remarks and Potential Future Avenues

The role of metabolic adaptation in CSCs remains open to investigation and could have significant implications in future therapeutics in different cancers, including breast cancer. Due to the interplay of different pathways, it becomes evident that a single therapeutic intervention could be limited by the metabolic flexibility of CSCs. Therefore, recognizing key pathways at play and blocking their feedback loop by devising a safe treatment combination may lead to a breakthrough in the future. In addition, the role of metabolic reprogramming in CSC dynamics and the interconversion between CSCs and non-CSCs in breast cancer needs to be elucidated. Future studies should also investigate the effect of signaling pathways on immune cell metabolism. The hedgehog pathway for example, is another CSC-related pathway and has been shown to suppress cytotoxic T-cells and anti-cancer immunity by modulating immune cell metabolism and the tumor microenvironment [186]. Effective combination therapies, therefore, should be able to inhibit CSC-associated pathways in the metabolic reprogramming of breast cancer while simultaneously promoting anti-tumor immunity.

## Figures and Tables

**Table 1:** Potential therapeutic strategies investigated in recent clinical trials for the treatment of breast cancer.

Pathway	Therapy	Subtype	Mechanism of Action	Clinical Trial ID
**YAP/TAZ**	Zoledronate	TNBC	Inhibits the mevalonate pathway via enzyme farnesyl diphosphate synthase [43].	NCT03358017
	Statin	Invasive BC	Inhibits the mevalonate pathway via HMG-coA Reductase [148].	NCT03971019
	Dasatnib	TNBC	Inhibits the nuclear translocation of YAP via its phosphorylation and degradation [45].	NCT02720185
	Alisertib	ER+ HER-2-	Inhibits Aurora A kinase, decreasing YAP stability [149].	NCT02187991
**HIF-1α**	Digoxin	Invasive BC	Inhibits PKM2-mediated transcription, indirectly reducing HIF-1α [150].	NCT01763931
	Irinotecan	Metastatic BC	Inhibits Topoisomerase1, shown to decrease HIF-1α expression [151].	NCT03562390
	Vorinostat	HER-2+ BC	Reduces nuclear accumulation of HIF-1α via HDAC inhibition [98].	NCT00574587
	Palbociclib	ER+ HER-2-	Disrupts HIF-1α stabilization via CDK inhibition [152].	NCT04247633
**PGC-1α**	TPST-1120	TNBC	Inhibits PPARα as a small molecule selective antagonist.	NCT03829436
	Neratinib	ER+ HER2-	Reduces ERRα-mediated gene expression via EGFR inhibition [153,154].	NCT04460430
**NF-kB**	Bortezomib	TNBC	Prevents nuclear translocation of NF-kB elements by proteosome inhibition [155].	NCT04265872
	IMX-110	TNBC	Decreases NF-kB signaling via nanoparticle encapsulation of curcumin [156].	NCT03382340
	Reparixin	TNBC	Attenuates NF-kB signaling by inhibition of upstream IL8 receptor CXCR1/2 [114].	NCT02370238
	N-acetylcysteine	Stage I BC	Reduces NF-kB activity [157].	NCT01878695
	Denosumab	Early BC	Inhibits receptor activator of NF-kB ligand (RANKL) [158].	NCT03324932
**Wnt signaling**	ETC-1922159	Advanced solid tumors	Prevents the processing of Wnt proteins as a porcupine inhibitor [159].	NCT02521844
	Eribulin	ER+/PR+ BC	Increases miR-195 expression, in turn decreasing expression of Wnt3a [160].	NCT03795012
	LGK974	TNBC	Targets porcupine inhibiting Wnt signaling [161].	NCT01351103
	SM08502	Advanced tumors	Reduces Wnt-mediated gene expression by interfering with alternative splicing [162].	NCT03355066
	PTK7-ADC	TNBC	Disrupts Wnt signaling by targeting protein tyrosine kinase 7 [163].	NCT03243331
	Vantictumab	Metastatic breast cancer	Inhibits Wnt-mediated signaling by targeting the frizzled receptors [164].	NCT01973309
**STAT3**	Sorafenib	Recurrent BC	Inhibits phosphorylation of STAT3 by enhancing phosphatase activity [165].	NCT00499525
	IMX-110	BC	Decreases STAT3 activity via nanoparticle encapsulation of curcumin [166].	NCT03382340
	TTI-101	Advanced BC	Specifically targets and binds to STAT3 preventing activation [167].	NCT03195699
	Ruxolitinib	TNBC	Inhibits STAT3 phosphorylation and downstream target genes [168].	NCT02876302
	Ritonavir	BC (all types)	Inhibits phosphorylation of STAT3 via HIV protease inhibition [169].	NCT01009437

Abbreviations: BC, breast cancer; TNBC, triple negative breast cancer; ER, estrogen receptor; HER-2, human epidermal growth factor-2; PKM2, pyruvate kinase isozyme; EGFR, epidermal growth factor receptor; HDAC, histone deacetylase; CDK, cyclin dependent kinase.
